# Effect of a Histone Demethylase Inhibitor on Equine Herpesvirus-1 Activity *In Vitro*

**DOI:** 10.3389/fvets.2018.00034

**Published:** 2018-03-12

**Authors:** Rebecca L. Tallmadge, Emilija Žygelytė, Gerlinde R. Van de Walle, Thomas M. Kristie, M. Julia B. Felippe

**Affiliations:** ^1^Equine Immunology Laboratory, Department of Clinical Sciences, College of Veterinary Medicine, Cornell University, Ithaca, NY, United States; ^2^Baker Institute for Animal Health, Cornell University, Ithaca, NY, United States; ^3^Molecular Genetics Section, Laboratory of Viral Diseases, National Institute of Allergy and Infectious Diseases, National Institutes of Health, Bethesda, MA, United States

**Keywords:** equine, horse diseases, Equine herpesvirus type 1, ganciclovir, histone demethylase inhibitor, epigenetic, viral load

## Abstract

Equine herpesvirus type 1 (EHV-1) is a ubiquitous and highly contagious pathogen that causes a range of disease severities with outbreaks of notable economic impact. Given the limitations in immune protection of current vaccines and the limited effectiveness of antiviral drugs on EHV-1 infections *in vivo*, improved treatment measures are needed to control disease. The use of drugs that alter the epigenetic state of herpes simplex virus genome has been shown to limit viral primary infection and reactivation both *in vitro* and *in vivo*. Therefore, we tested the hypothesis that maintaining a repressive epigenetic state on the EHV-1 genome in the host equine cell would decrease viral load during lytic infection. Equine fetal kidney cells (EFKCs) or isolated peripheral blood leukocytes were treated *in vitro* with (a) the nucleoside analog ganciclovir; (b) the histone demethylase inhibitor OG-L002; (c) both ganciclovir and OG-L002; or (d) dimethyl sulfoxide (DMSO, vehicle control); and then infected with a clinical EHV-1 isolate. Treatment of EFKCs with ganciclovir (mean 22.3 DNA copies per cell, *p* = 0.0005), OG-L002 (mean 25.6, *p* = 0.005) or both ganciclovir and OG-L002 (mean 7.1, *p* = 0.0001) resulted in decreased EHV-1 viral load at 24 h post-infection (hpi) in comparison with DMSO (mean 42.0), with greater impact using the combined treatment. Further, EHV-1 gene expression at 3 hpi decreased when EFKCs were infected in the presence of ganciclovir (*p* = 0.04) and combined treatment of ganciclovir and OG-L002 (*p* = 0.0003). In contrast, under similar conditions, neither ganciclovir nor OG-L002 suppressed EHV-1 infection in leukocytes. Differences between cell types, drug penetrance, or drug turnover, may have contributed to the distinct effects observed in this study.

## Introduction

Equine herpesvirus 1 (EHV-1) is a serious threat to horses worldwide (1). EHV-1 outbreaks have emphasized the potential effects of this virus including: (1) severe respiratory disease, neurological disease, abortion, or death; (2) the highly contagious nature of the virus; (3) incomplete protection from vaccination and antiviral therapy; and (4) substantial economic costs for the equine industry (2–11). The prevalence of EHV-1 among 4,228 horses with fever, respiratory signs, and/or neurological deficits was 2.7% in the USA between 2008 and 2014 (12). The incidence of EHV-1-induced neurological disease was reported increased over the last decade internationally (13–15). Therefore, the global equine industry is compelled to identify more effective EHV-1 control measures and treatments.

Equine herpesvirus 1 infection is initiated by inhalation of infectious virus, whereupon the virus enters epithelial cells of the upper respiratory tract and replicates, leading to viral shedding *via* nasal discharge, and further spreading of the infection (16). Fever and respiratory clinical signs may ensue, although some horses experience subclinical shedding (5). EHV-1 upper respiratory tract entry facilitates infection of leukocytes, enabling the virus to circulate and infect endothelial cells of the central nervous system, leading to myeloencephalopathy; or reach the pregnant uterus, causing late-term abortion (17–19).

Although much effort has been invested in improving EHV-1 vaccines to prevent disease and curtail virus spread, current vaccines offer limited protection from infection or reactivation of the virus. Vaccines presently on the market have been shown to suppress EHV-1 disease and shedding but may not limit viral load. The level of protection against EHV-1-induced neurological disease by vaccination is unclear, and not claimed by any of the vaccine manufacturers (20–22). Further, natural exposure induces immune protection that can be as short as 3–6 months (20–23). Care of infected animals includes administration of antiviral drugs and supportive therapies (6, 24, 25). During EHV-1 infection, antiviral treatment using synthetic nucleoside analogs shows promising efficacy in cell culture models, but treatment of ponies with an experimental EHV-1 infection has been less effective (25–28). Currently used antiviral drugs are only active during lytic infection as they interfere with replication of the viral genome and, as such, they have no effect on latent EHV-1.

After EHV-1 infects the horse cell, the EHV-1 DNA genome is released into the nucleus to eventually produce more infectious EHV-1. A cost of this strategy is that the EHV-1 DNA is now subjected to the host cell’s gene regulation machinery. Recent work with human herpes simplex virus (HSV1) has demonstrated that the viral genome becomes susceptible to host-mediated epigenetic regulation, including the assembly and modulation of host-derived histones on the viral DNA genome (29–31). Subsequently, posttranslational modifications of the histone tails either permit or repress viral gene expression (29, 32, 33). Remarkably, maintaining a repressive epigenetic state of the HSV1 DNA suppressed viral gene expression during lytic infection, and suppressed reactivation from latency *in vitro* and *in vivo* (33–35). In these studies, the repressive state was preserved by preventing the removal of methyl groups from lysine 9 of histone 3 (H3K9) with the use of compounds that block the activity of the lysine-specific demethylase 1 (LSD1) protein [e.g., tranylcypromine, a monoamine oxidase inhibitor (MAOI); or a novel selective LSD1 inhibitor, OG-L002] (33–35). Because LSD1 is not the only protein that modulates histone methylation, LSD1 inhibition is not expected to have global effects. Further, the use of a histone demethylase inhibitor in conjunction with standard antiviral therapy in experimental HSV1 infection exerted a synergistic reduction of active infection, and limited reactivation from viral latency (33).

Regulating EHV-1 by maintaining a repressive epigenetic state would offer a new strategy to combat both lytic and latent EHV-1 infections. Although the extent of epigenetic regulation of the EHV-1 genome has not been determined to date, recent work showed that enforcing a permissive epigenetic state accelerates EHV-1 protein expression and induces productive infection (36). This finding suggests that EHV-1 DNA is subject to epigenetic regulation in horse cells and EHV-1 gene expression can be modulated by altering histone modifications. Given these data, we hypothesize that maintaining a repressive epigenetic state of the EHV-1 genome in the host equine cell would decrease viral load during lytic infection. To test this hypothesis, we investigated how histone tail hypermethylation altered EHV-1 lytic infection of permissive equine cells *in vitro*.

## Materials and Methods

This study was carried out in accordance with the recommendations of Institutional Animal Care and Use Committee for the use of vertebrates in research, and the protocol approved by the Cornell University Center for Animal Resources and Education.

### EHV-1 Isolate

Our laboratory isolated the EHV-1 virus from a naturally infected, neurologically affected research horse at Cornell University in 2005 using permissive rabbit kidney 13 cell cultures [titer of 1 × 10^7^ plaque forming units (PFU)/mL], and this isolate was confirmed to harbor the ORF30 G2254 allele by PCR performed at the Cornell University Animal Health Diagnostic Center.

### Cells, Treatments, and *In Vitro* Cultures

Primary equine fetal kidney cells (EFKCs) were isolated in our laboratory (37). The EFKCs were frozen in cell freeze media and stored in liquid nitrogen. Upon thawing, passage 5 of EFKCs were seeded into 12-well tissue culture plates in DMEM-F12 medium containing 10% fetal bovine serum, and 1× antibiotics–antimycotics (ThermoFisher Scientific, Waltham, MA, USA) until they reached 80–90% confluence before treatment and EHV-1 infection.

Blood samples were collected by jugular venipuncture into vacutainers containing heparin sulfate from three research healthy adult horses (two Warmblood mares and one Pony gelding, age range 14–20 years) from the Cornell Equine Park, Ithaca, NY, USA. Peripheral blood mononuclear cells were isolated using a previously described protocol of Ficoll-Paque density centrifugation (38).

We selected OG-L002 for these experiments rather than tranylcypromine to avoid unintended inhibition of other MAOI targets in the equine cell. The nucleoside analog ganciclovir was used as a model antiviral drug as *in vitro* EHV-1 studies demonstrated that ganciclovir was the most potent of six antiviral drugs with the lowest, non-toxic effective concentration (28). A combined epigenetic and antiviral treatment was also included in our experimental design based on observed synergistic effects when dual LSD1 inhibitor and nucleoside analog treatments were studied in HSV1 infection *in vivo* (34).

### Evaluation of Treatment Toxicity *In Vitro*

**Figure 1:**
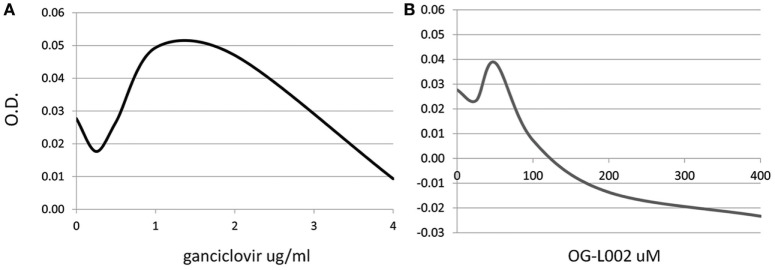
Dose-dependent cytotoxic effect of treatment. Cell viability of equine leukocytes was tested after 24 h of treatment with different concentrations of ganciclovir **(A)** or OG-L002 **(B)** using the MTT assay. Results indicate best-fit curve of mean values obtained from triplicate experiments at 540 nm optical density (OD).

To determine whether the ganciclovir or histone demethylase inhibitor treatments exerted a cytotoxic effect on the cultured cells, a range of ganciclovir (0.25–4.0 µg/mL) or OG-L002 (25–400 µM) concentrations were tested on equine leukocytes (0.25 × 10^6^ cells/well) in triplicate for 24 h of culture in RPMI medium with 10% fetal bovine serum and 1X antibiotics/antimycotics (ThermoFisher Scientific, Waltham, MA, USA). Cell metabolic activity *via* enzymatic reduction of the tetrazolium dye MTT to its insoluble formazan was measured in order to assess cell viability (ATCC, Manassas, VA, USA). Based on the results, the concentrations used in the subsequent infection experiments were 50 µM OG-L002 and 1 µg/ml ganciclovir (Figure 1). Comparable concentrations have been used in other studies without evidence of cytotoxicity (28, 35).

Equine fetal kidney cell (1 × 10^5^ cells/well) or leukocyte (5 × 10^5^ cells/well) cultures were pretreated for 4 h with one of the following: (1) LSD1 inhibitor OG-L002 (50 µM, Selleck Chemicals, Houston, TX, USA); (2) nucleoside analog ganciclovir (1 µg/mL, Sigma-Aldrich Corporation, St. Louis, MO, USA); (3) both OG-L002 and ganciclovir at their respective concentrations; or (4) 0.005% dimethyl sulfoxide (DMSO, vehicle control, drug diluent). Cell cultures were then infected with 7.5 × 10^3^ PFUof EHV-1 for 1 h, followed by replacement of viral inoculum with media containing the respective compounds. Replicate wells were included in each experiment and independent experiments were performed two or three times. Cells were harvested at 3 (for RNA isolation and viral gene expression analysis) or 24 h post-infection (hpi) (for DNA isolation and viral load analysis). For kinetic analysis of gene expression, samples were harvested at 1, 3, 6, 12, and 24 hpi without treatment.

### Viral Load Quantification

Twenty-four hours after respective treatment and EHV-1 infection, media from cultured EFKC or leukocytes were removed and cells directly lysed using a Quick-gDNA MiniPrep kit (Zymo Research, Irvine CA, USA). The DNA isolation was performed as directed by the manufacturer immediately after lysis or after homogenized samples were stored at −80°C. Quantitative PCR (qPCR) was performed to measure viral loads and equine cell genomic DNA using primers to the viral gB or the cellular SP1 genes, respectively (24). Table 1 shows the primer sequences used in this study. Reactions were performed in triplicate with iTaq Universal SYBR Green Supermix on a CFX96 Real Time System, including no template controls (Bio-Rad Laboratories, Inc., Hercules, CA, USA). The number of EHV-1 copies per cell was calculated by dividing the number of gB copies by the number of SP1 copies, and then multiplying that value by two to account for two SP1 alleles per equine genome.

**Table 1:** Primers used in SYBR quantitative RT-PCR assays.

Gene	Forward primer (5′–3′)	Reverse primer (5′–3′)
EHV-1 IE	GCTGGGCGGACACATAGT	ACCTCTCCTGAACACGATGG
EHV-1 UL5	CGACGATGAGAGGTAGCA	GCACCAGAGACCCTATCA
EHV-1 gB	TATACTCGCTGAGGATGGAGACTTT	TTGGGGCAAGTTCTAGGTGGTT
SDHA	CAGACGATTTATGGAGCAGAGG	CTGGATGGGCTTGGAGTAAT
SP1	GGTCATACTGTGGGAAAC	GGTCTATTACTATTTCTTCCTTC

### Gene Expression Quantification

Media from cultured EFKC cells were removed and cells directly lysed using Quick-RNA microprep (Zymo Research, Irvine, CA, USA). The RNA isolation was performed immediately after lysis or after homogenized samples were stored at −80°C. After isolation, RNA was treated with DNase I to degrade contaminating equine genomic and viral DNA (ThermoFisher Scientific, Waltham, MA, USA). Quantitative RT-PCR assays were designed and validated for EHV-1 detection with iTaq Universal SYBR Green One-Step kit for the EHV-1 immediate early gene (IE), an early gene (UL5), and a late gene (gB) (Table 1). Assay specificity was confirmed using uninfected cell controls and each gene was cloned to generate a standard curve. Quantitative RT-PCR was performed with these samples to identify an equine reference (housekeeping) gene. After comparing expression of six reference genes (beta-actin, c-MYB, GAPDH, SDHA, SP1, and SLC25A44), SDHA was chosen as the expression was consistent, and the primers did not amplify equine genomic DNA (data not shown; SDHA and SLC25A44 primer sequences provided by D. Miller and D. F. Antczak, Cornell University College of Veterinary Medicine, Ithaca, NY, USA). Reactions were performed in triplicate with the iTaq Universal SYBR Green One-Step kit on a CFX96 Real Time System, including no reverse-transcriptase controls and no template controls (Bio-Rad Laboratories, Inc., Hercules, CA, USA). Relative EHV-1 gene expression was determined by calculating the difference between the EHV-1 gene, and horse SDHA cycle threshold values for each sample (normalization). For kinetic analysis, gene expression at 1 h was set to 100%, and that of later time points was determined relative to this value with the ddCt method. For experiments, expression of the IE gene in DMSO-treated EHV-1-infected cells was set to 100%, and expression in treated cells determined relative to this value.

### Statistical Analysis

The Shapiro–Wilk normality test was employed to determine whether data fit a normal distribution. When data fit a normal distribution, ANOVA including Tukey’s multiple comparisons test was performed to assess effect of treatments, and mean values were provided. Alternatively, when data did not fit a normal distribution, analyses were conducted with Kruskal–Wallis tests including Dunn’s multiple comparisons test, and median values were provided. All statistical tests were performed with GraphPad Prism (GraphPad Prism v6.05 for Windows, San Diego, CA, USA). A *p*-value ≤0.05 was considered significant.

## Results

The objective of this work was to measure the effect of inhibition of the histone demethylase LSD1 on EHV-1 lytic infection *in vitro*. Adherent primary EFKCs or leukocyte cultures were treated and infected, and the resulting EHV-1 viral load and gene expression quantified.

### Effect of Treatment on EHV-1 Viral Load During Lytic Infection of Primary EFKCs

**Figure 2:**
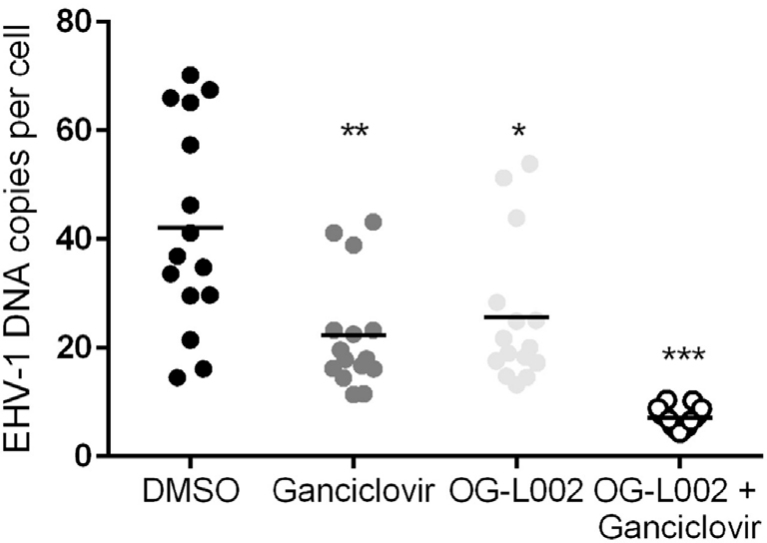
Effect of treatment on equine herpesvirus type 1 (EHV-1) viral load during lytic infection of primary equine fetal kidney cells. At 24 hpi, reduced EHV-1 DNA copies per cell were detected in the presence of ganciclovir (**p = 0.0005) or OG-L002 (*p = 0.005), and markedly when both treatments (***p < 0.0001) were applied simultaneously in comparison with DMSO control vehicle. Combined treatment decreased the number of EHV-1 DNA copies in comparison to treatment with ganciclovir alone (*p* = 0.01) or OG-L002 alone (*p* = 0.001). Lines represent mean values.

To quantify EHV-1 viral load in the presence of ganciclovir and/or OG-L002 during lytic infection of primary EFKC *in vitro*, DNA was harvested at 24 hpi and qPCR performed for EHV-1 gB and equine SP1 targets. Viral DNA loads per cell were calculated as described previously. In comparison to the DMSO vehicle control (mean 42.0, 14.6–70.2), the number EHV-1 DNA copies per cell was reduced (*p* = 0.0005) in the presence of ganciclovir alone (mean 22.3, range 11.4–43.2), OG-L002 alone (mean 25.6, range 13.3–53.9, *p* = 0.005), and when treatment included both ganciclovir and OG-L002 (mean 7.1, range 4.4–10.4, *p* < 0.0001) (Figure 2). Further, combining the treatments decreased the number of EHV-1 DNA copies in comparison to treatment with ganciclovir alone (*p* = 0.01) or OG-L002 alone (*p* = 0.001), whereas there was no statistical difference between ganciclovir alone and OG-L002 alone (*p* = 0.9).

### Kinetic Analysis of EHV-1 Gene Expression During Lytic Infection of Primary EFKCs

**Figure 3:**
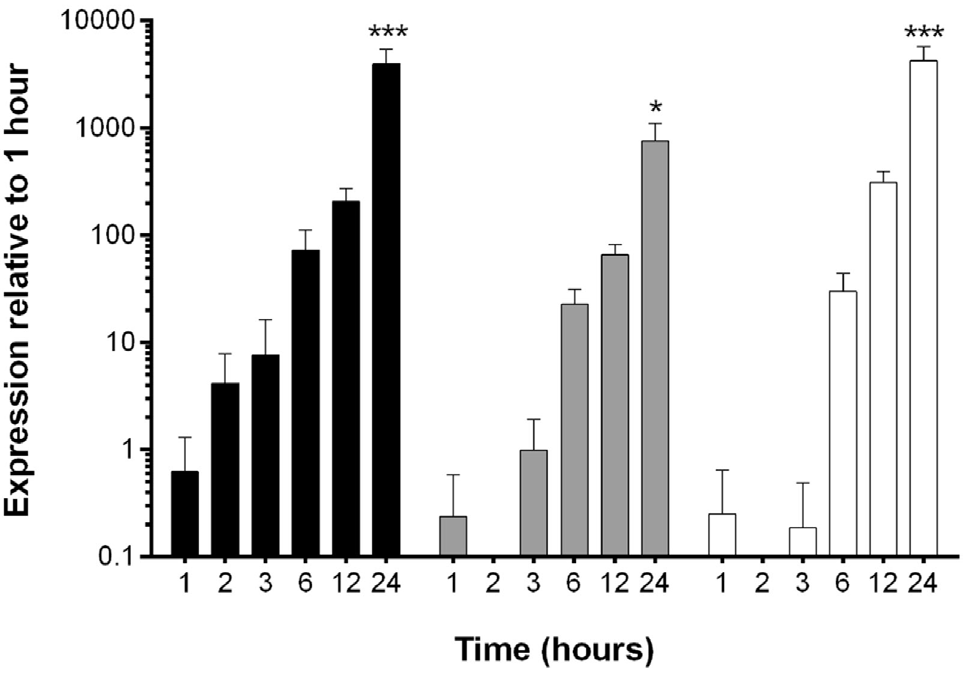
Kinetic analysis of equine herpesvirus type 1 gene expression during lytic infection of primary equine fetal kidney cells (EFKCs). Expression of the IE gene was detected in EFKCs starting at 1 hpi (black bars), whereas expression of UL5 (gray bars) and gB (white bars) genes was not consistently detected until 3 and 6 hpi, respectively. Data are shown as mean plus SD. Statistical significance is indicated by asterisks and denotes an increase at 24 hpi compared to all earlier time points (*p < 0.05, ***p < 0.0001).

The reduction in EHV-1 viral load in EFKCs observed 24 h after treatment with OG-L002 suggested that this epigenetic modulator inhibited EHV-1 activity. To further study this effect, we measured how OG-L002 treatment altered EHV-1 gene expression soon after infection. First, a kinetic study without treatment of EFKC was undertaken to assess EHV-1 immediate early (IE), early (UL5), and late (gB) gene expression from 1 to 24 hpi. At 1 hpi, expression of the EHV-1 IE gene was close to the lower limit of detection, steadily increased by 2 hpi, and became robust by 3 hpi (Figure 3). Expression of UL5 gene followed a similar pattern, but consistent detection was measured at 3 hpi. The gB gene was abundantly expressed at 6 hpi and beyond. At 24 hpi, IE, UL5, and gB gene expressions were increased (*p* < 0.05) in comparison to all earlier time points. Since the EHV-1 immediate early and early genes were consistently detected at 3 hpi, this time point was selected for RNA harvesting and analysis of treated samples.

### Effect of Treatment on EHV-1 Gene Expression during Lytic Infection of Primary EFKCs

**Figure 4:**
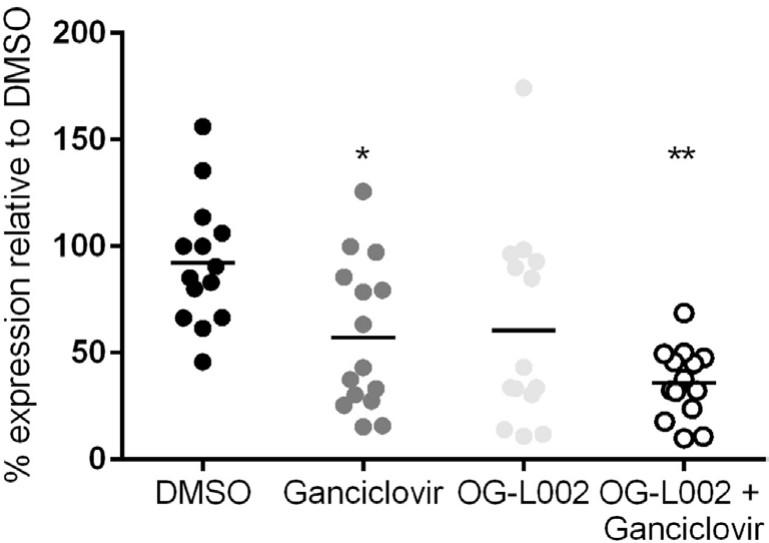
Effect of treatment on equine herpesvirus type 1 (EHV-1) IE gene expression during lytic infection of primary equine fetal kidney cells. EHV-1 IE gene expression was decreased in the presence of ganciclovir (*p = 0.04) and combined treatment of ganciclovir and OG-L002 (**p = 0.0003) at 3 hpi, when compared with DMSO control vehicle. Lines represent mean values.

Experiments were then undertaken to test the effect of ganciclovir and/or OG-L002 treatment on EHV-1 IE gene expression during lytic infection of primary EFKC *in vitro*. At 3 hpi EHV-1 infection, RNA was isolated and qRT-PCR was performed for the EHV-1 IE gene and equine SDHA reference gene. Decreased (*p* = 0.04) EHV-1 IE gene expression was observed with ganciclovir treatment (mean 57.2, range 15.2–125.7) in comparison to DMSO vehicle control (mean 92.2, range 45.7–156.2) (Figure 4). Treatment with OG-L002 trended toward a reduction (*p* = 0.08) in IE gene expression (mean 60.6, range 10.9–174.4); data analysis performed when an outlier was removed in the OG-L002 resulted in significant statistical difference between OG-L002 and DMSO vehicle control (*p* = 0.005). Combined treatment of both ganciclovir and OG-L002 resulted in a synergistically decreased (*p* = 0.0003) IE gene expression (mean 35.9, range 9.8–68.7) when compared to DMSO vehicle control. Further, combining the treatments did not result in statistically significant differences in EHV-1 IE gene expression when compared to ganciclovir alone (*p* = 0.3) or OG-L002 alone (*p* = 0.2) or when ganciclovir alone was compared to OG-L002 alone (*p* = 1.0).

### Effect of Treatment on EHV-1 Viral Load During Infection of Equine Peripheral Blood Leukocytes

**Figure 5:**
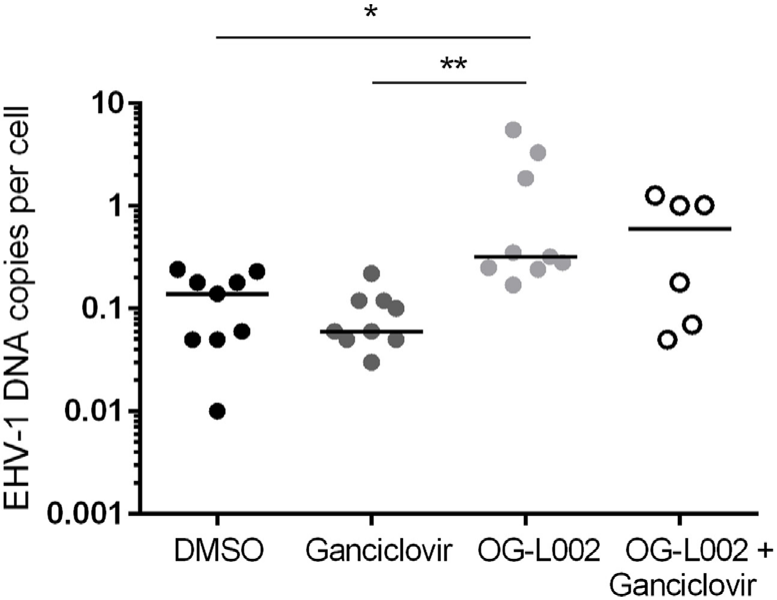
Effect of treatment on equine herpesvirus type 1 (EHV-1) viral load during infection of equine leukocytes. At 24 hpi, increased EHV-1 DNA copies per cell was detected in the presence of OG-L002 when compared with DMSO vehicle control (*p = 0.02) and ganciclovir (**p = 0.002). There was no difference (*p* > 0.05) in EHV-1 viral load between DMSO vehicle control, ganciclovir or OG-L002 plus ganciclovir treatments. Note that *y*-axis has a log10 scale. Lines represent median values.

Determining the effect of histone demethylase inhibitor treatment on EHV-1 infection of leukocytes is relevant to the clinical disease condition because leukocytes are integral to the systemic spread of EHV-1 and harbor latent EHV-1 (17, 26, 39–41). Leukocytes were pretreated with ganciclovir, OG-L002, both ganciclovir and OG-L002, or with DMSO vehicle control, and then infected with EHV-1 as described previously. DNA was harvested at 24 hpi, and qPCR performed to quantify the EHV-1 DNA copies per cell. In contrast to the experiments performed with EFKC infection, the presence of ganciclovir (median 0.06, range 0.03–0.2) did not change (*p* > 0.05) the number of EHV-1 copies per leukocyte compared to DMSO vehicle control (median 0.14, range 0.01–0.24). Under these culture conditions, OG-L002 treatment (median 0.32; range 0.17–5.5) did not suppress EHV-1 viral load in leukocytes when compared with DMSO vehicle control (*p* = 0.02), or with ganciclovir (*p* = 0.002) treatment (Figure 5). Treatment of leukocytes with both ganciclovir and OG-L002 (median 0.59, range 0.05–1.3) yielded an EHV-1 viral load equivalent (*p* > 0.05) to DMSO vehicle control, ganciclovir alone (*p* = 0.2) and OG-L002 alone (*p* = 1.0). Viral replication in leukocytes without treatment was confirmed in independent experiments based on a 5.9-fold increase in IE expression at 24 hpi when compared to values at 3 hpi (*p* = 0.049).

## Discussion

This work assessed the potential of the LSD1 inhibitor OG-L002 to limit EHV-1 viral load and gene expression during lytic infection *in vitro*. When used individually, OG-L002 inhibited EHV-1 activity in EFKCs to levels similar to that of ganciclovir treatment. When used in combination, a synergistic effect of ganciclovir and OG-L002 was measured. This observation reinforces the premise that these drugs inhibit viral replication through independent mechanisms, and could be an effective strategy to limit EHV-1 infection. However, under the culture conditions described herein, neither ganciclovir nor OG-L002 treatment of leukocytes suppressed EHV-1 viral load. Studies on the effect of compounds that alter epigenetic modifications on leukocytes have not been published before, and drug dosage or cell penetrance may account for the cell-specific impacts of these compounds on fetal kidney cells relative to equine leukocytes. The lack of EHV-1 inhibition by ganciclovir treatment in leukocytes was also unexpected, particularly in contrast to the results of EFKC cultures. These findings further suggest that differences between the cell types may be due to either cell bioavailability and/or length of exposure *in vitro*. Studies demonstrating the efficacy of ganciclovir in controlling EHV-1 in equine embryonic lung cells, equine dermal cells, pig kidney cells, or rabbit kidney cells rather than leukocytes have been reported (28, 39–41). Of note, ganciclovir treatment of human lymphoblastoid cells *in vitro* showed minimal antiviral effect when used for less than 7 days, even at high doses of 20 µg/mL; and, significant effects of ganciclovir treatment were observed at 14 days (42). In addition, the efficacy of antiviral drugs *in vivo* has not been consistent with *in vitro* findings, partly due to bioavailability or differential cell sensitivity (43–45). Unlikely these findings were due to lower efficiency of viral replication in leukocytes in comparison to kidney cells, since viral IE expression increased at 24 hpi.

With respect to the cell type dependence of the LSD1 inhibitor OG-L002, there is also the potential that tissue-specific expression or activities of LSD1 isoforms may have resulted in the lack of impact in leukocytes. Several LSD1 isoforms are generated by alternative splicing and these can differ in substrate specificities (H3K4 versus H3K9) and coregulatory protein interactions, resulting in differential gene regulation (46). Nevertheless, inhibition of histone demethylases, including LSD1, have been shown to inhibit HSV, human cytomegalovirus, adenovirus type 5, and varicella zoster virus (VZV) in primary fibroblasts *in vitro*, and to suppress HSV lytic infection and reactivation in multiple animal models (33–35).

The principal hypothesis of this study was based on previous HSV studies, in which promoting or maintaining an epigenetic repression of the virus suppressed viral infection and reactivation from latency. Therefore, it was anticipated that epigenetic suppression of EHV-1 would decrease viral load during lytic infection and prevent reactivation. Because effects were seen on the transcription of the EHV-1 IE gene in EFKC, the histone demethylase inhibitor OG-L002 was likely to be functioning as expected. In support of this observation, we identified a putative enhancer core element in the EHV-1 genome that may serve as the binding site for the protein complex needed for LSD1 activity, with the motif TAATGAGAT at positions 119,838–119,846 of the EHV-1 genome sequence NC_001491. For HSV and VZV IE gene expression, cellular transcription factors and proteins in the virion recruit host cellular transcriptional coactivator host cell factor-1, LSD1, and JMJD2 to bind the enhancer core element upstream of the IE gene (33). The presence of the enhancer core motif upstream of EHV-1 ORF64 and near other transcription factor binding motifs (SP1, CCAAT, and TATA) supports the likelihood that epigenetic mechanisms similar to those described for HSV1 also regulate EHV-1.

Additional experiments are required to confirm the inhibition of histone demethylation in the equine system, and optimization of the type of drug and dosage for different cell types. Inhibitors of other proteins in the same pathway also exert antiviral activity, and it would be of interest to investigate their impact on EHV-1 infection (47). If testing other compounds would show an effect on viral load in equine leukocytes, the use of respective drug inhibitor controls and additional experiments testing mechanisms of action should be pursued. The use of compounds that alter epigenetic modifications in the host cell rather than targeting viral protein(s) is advantageous in that it is not likely to elicit resistant viral strains. Identifying treatments that simultaneously impair EHV-1 lytic infection and reactivation from latency through a mechanism complementary to current antiviral drugs would be of great value in gaining control of EHV-1 disease severity and spread.

In conclusion, this study showed a favorable effect of the histone demethylase inhibitor OG-L002 in controlling EHV-1 load and gene expression in equine kidney cells, and these results support our hypothesis that maintaining a repressive epigenetic state on the EHV-1 genome in the host equine cell decreases viral load during lytic infection. However, this same effect was not observed in EHV-1 infected equine leukocytes, and additional compounds that alter epigenetic status should be tested.

## Ethics Statement

This study was carried out in accordance with the recommendations of Institutional Animal Care and Use Committee for the use of vertebrates in research, and the protocol approved by the Cornell University Center for Animal Resources and Education.

## Author Contributions

RT: designed and prepared grant proposal that funded study, including hypothesis, specific aims, and methods; performed molecular experiments; compiled data; performed statistical analyses; and prepared manuscript draft. EŽ: performed part of the molecular experiments; compiled data; performed statistical analyses; participated in discussions; and edited manuscript draft. GW and TK: provided expertise, input and editing of grant proposal preparation (coinvestigator), including methods and experimental design; discussed results and conclusions; and edited manuscript draft. MF: designed and prepared grant proposal that funded study, including hypothesis, specific aims, and methods; performed cellular experiments; compiled data; and edited manuscript draft.

## Conflict of Interest Statement

The authors declare that the research was conducted in the absence of any commercial or financial relationships that could be construed as a potential conflict of interest.
